# Linking physical environment in physical education classes and adolescents' life skills: evidence from Chinese secondary schools

**DOI:** 10.3389/fpubh.2026.1844695

**Published:** 2026-07-23

**Authors:** Jiahao Wang, Lan Pang, Wenhao Xue, Xinyi Xie, Rong Fan

**Affiliations:** Department of Physical Education, China University of Geosciences Wuhan, Wuhan, China

**Keywords:** adolescents, learning engagement, life skills, mediation model, physical education environment

## Abstract

**Background:**

Adolescents' life skills(LS) development has become increasingly important in China's ongoing education reform, but the associations between the physical environment of physical education classes (PEOPEC), physical education class learning engagement (PECLE), and LS remain insufficiently understood. Grounded in ecosystem theory and achievement goal theory, a mediation model of “PEOPEC—PECLE—LS” was constructed and validated to explore the pathways through which the implicit educational mechanisms of physical education curricula operate.

**Methods:**

Using questionnaire data from 504 secondary school students in the central and eastern regions of Hubei Province, this study tested the proposed model using Pearson correlation analysis and bootstrap-based mediation analysis.

**Results:**

indicated that students' perceived PEOPEC was significantly and positively associated with all eight dimensions of LS, including teamwork and goal setting. Furthermore, PECLE partially statistically mediated these associations.

**Discussion:**

It is recommended that efforts begin with optimizing PEOPEC by systematically improving teaching spaces, equipment allocation, and situational design quality. Such measures may be relevant for supporting students' learning engagement and realizing the educational value of PE curricula.

## Introduction

As basic education increasingly emphasizes students' transferable competencies and holistic development, an important question remains: the extent to which learning in physical education classes can be transferred to students' daily lives and translated into abilities that can be applied beyond the classroom (1). On the one hand, there is a disconnect between physical education teaching content and students' daily lives, with students participating passively in class while finding it difficult to realize skill transfer in life (2). In addition, there remains a marked gap between the development of some students' life skills (LS) and the goals of core competencies (3). How to improve the transfer from physical education learning to LS has become an important issue in current educational reform (4). Against this background, the perspective of LS provides a more operational point of entry.

LS are functional skills that are developed and applied by individuals in specific contexts and include eight dimensions: teamwork, goal setting, social skills, issue decision, emotional skills, leadership, time management, and communication skills (5). LS not only help adolescents cope with changing social situations and enhance their adaptive competitiveness, but are also broadly aligned with the three core competency domains of “cultural foundation, self-directed development, and social participation,” thereby providing important support for students' holistic development (6). Therefore, systematically integrating LS into physical education curricula is not only consistent with the goals of educational modernization, but also provides an important pathway for implementing the educational goal of promoting students' development in moral, intellectual, physical, aesthetic, and labor-related domains.

Schools are an important setting for students' daily learning and socialization, and the learning environment, as an external condition, is closely related to individuals' cognition, behavior, emotions, and learning engagement (7). Compared with other subjects, physical education has dynamic, contextual, and practice-oriented characteristics, and thus holds unique value in the cultivation of LS. Research has shown that students are often able to experience life-related situations involving cooperation, stress coping, self-expression, and decision making more directly through exercise, which may support the development of their key competencies (8). These teaching practices are supported by physical environmental factors in physical education classes, including the spatial layout of teaching venues, the quality of facilities and equipment provision, and safety provisions (9). A favorable physical environment may be directly related to students' classroom experiences and may also be indirectly associated with LS through psychological processes such as learning motivation, emotional arousal, and social interaction.

Based on this, the present study focuses on the current status of PEOPEC, examines its associations with students' LS, and further investigates whether learning engagement statistically mediates these associations.

### Life skills (LS)

LS are closely related to the social and living environment, and their acquisition and development are important for the holistic development of adolescents. This study defines LS as functional skills that individuals develop and apply in specific contexts to cope with demands arising from family, study, sports, community, and work, and that can be transferred to other contexts (10). Achievement goal theory holds that individuals' Physical Education Classes Learning Engagement (PECLE) is related to students' pursuit of skill improvement and ability development; when students perceive a classroom environment as appropriately challenging and safe, they are more likely to form a mastery goal orientation and show greater willingness to learn, while a favorable learning environment may support students' beliefs in effort and their learning initiative (11). Behavior in physical education classes is based on individual thinking and group coordination, which requires students to possess a certain degree of leadership and teamwork skills to respond to various situations that arise during physical education classes; the classroom is also an important setting for developing students' communication skills and emotional regulation, and thus LS are also manifested in physical education classes (12, 13).

Adolescents face multiple challenges in the course of development, some of which may threaten their healthy development; especially for socially disadvantaged groups, cultivating LS through exercise may become an important means of future social adaptation (14). As school physical education provides an important setting for LS cultivation, it should encourage adolescents to reflect on their exercise experiences to support the development of LS (15). Physical environment of physical education classes (PEOPEC), PECLE, and LS development may be interrelated and may jointly contribute to the effectiveness of physical education learning and students' overall competencies.

### Physical environment of physical education classes (PEOPEC)

In the field of physical activity, one of the important dimensions of the ecological model is the physical environment (7). School sports facilities provide essential material conditions for physical education teaching, and their adequacy may be related to the scope of teaching content and teaching quality (16). The school environment is regarded as an important contextual correlate of adolescents' physical activity (17). Its optimization can enhance students' participation in physical activity (18). As a micro-level component of the school environment, PEOPEC is a key component of the physical education teaching environment and is related to students' overall perceptions of and responses to the classroom, encompassing all material conditions required for the implementation of physical education teaching (19). The design of the physical environment has been associated with students' level of PECLE, classroom participation, and learning outcomes (20). In environmental psychology, theories of prosocial behavior suggest that environmental interventions may be associated with individual behavior and exploratory initiative, indicating the important role of environmental plasticity in behavioral development (21).

Research has shown that the relative contribution of physical education class venues and equipment to students' learning satisfaction is second only to teachers' instructional competence (22). The greatest beneficiaries of the successful intelligent transformation of sports venues will be students (23). Diversified activity spaces are an excellent instrument for strengthening students' aesthetic literacy and innovative awareness, which reflects a value distinct from explicit educational factors such as teacher behavior and teacher–student interaction. For example, increasing public sports facilities, updating sports equipment, and optimizing sports grounds may be associated with higher overall physical activity levels and greater social interaction (24). The physical environment has been reported to be an important correlate of physical activity levels, second only to individual and social factors; facility accessibility, diversity, and novelty are among the factors associated with exercise behavior (25). At the theoretical level, Bronfenbrenner's microsystem theory posits that the microsystem is the environment in which children directly interact and establish relationships, including family, school, and peers, emphasizing that while the environment shapes the individual, the individual also shapes the environment. The school classroom environment includes physical conditions (such as classroom layout and resources) and social interaction; an organized and well-laid-out teaching environment may be associated with student interaction, classroom inclusiveness, and students' learning experiences and outcomes (26). Therefore, optimizing PEOPEC may be relevant to students' PECLE and multiple dimensions of LS, as well as to the attainment of physical education objectives.

### Physical education class learning engagement (PECLE)

Engagement is commonly defined as a positive, fulfilling, and highly task-related psychological state, with its core characteristics including vitality, dedication, and concentration (27). Among these, vitality refers to an individual being full of energy and possessing high adaptability in learning or work; dedication is reflected in enthusiasm for the task, a sense of meaning, and a sense of challenge; concentration is manifested as a psychological state of full attention and immersion. Although there is still no unified definition of engagement in physical education learning, existing studies have shown that, in educational settings, learning engagement is a malleable psychological state, and physical education courses may support students' active participation (28). Drawing on the literature, this study defines PECLE as a sustained psychological and behavioral state in which students actively strive to master knowledge and skills in physical education classes and show a high degree of attention to and participation in learning tasks.

The theory of engagement was first proposed by Mosher and McGowan (29) in their study of learner dropout, and subsequent research has paid greater attention to its multidimensional structure. This study adopts its two dimensions, cognitive engagement and behavioral engagement, as theoretical support. In the context of sports learning, as an external contextual factor, the physical environment has been associated with students' behavioral motivation and depth of engagement and may provide a contextual basis for adolescents' competency development, particularly for marginalized groups in specific environments (30). Research has also indicated that, teacher support has been associated with higher levels of learning engagement, peer interaction, and communication among high school students during the learning process (31). From the cognitive perspective, learning engagement is closely related to cognitive participation, and students engage in deeper cognitive processing when interacting with specific learning tasks and environments (32). Cognitive participation is context-dependent, and the complexity of the environment may be related to students' participation behavior. Higher levels of learning engagement may be associated with more favorable skill mastery and ability development.

Through the above variable descriptions and theoretical explanations, it can be argued that when students perceive and interpret external environmental stimuli, the physical environment may be associated with students' PECLE, which may in turn be associated with LS development. Microsystem theory provides the model with a foundation for contextual embedding and environmental explanation, while achievement goal theory provides a framework for understanding motivation and behavioral responses. Motivational processes are embedded in environmental contexts and therefore cannot be separated from the microsystem. Together, the two provide a theoretical basis for examining the hypothesized association pattern of “PEOPEC–PECLE–LS”. Based on the above analysis, this study proposes the following hypotheses: H1: PEOPEC is positively associated with LS development; H2: the three dimensions of PECLE are positively associated with LS development; H3: PECLE has a partial mediating effect between PEOPEC and the development of LS.

## Method

### Participants

Participants were recruited from two junior secondary schools and two senior secondary schools in the central and eastern regions of Hubei Province, China. The selected schools included urban and county-level schools, public and private schools, and key and regular schools, providing a heterogeneous school sample. Three intact classes were randomly selected from each school. Data were collected between April 20, 2024, and August 16, 2024, covering the routine teaching period of the spring and summer terms.

A total of 531 questionnaires were distributed and collected. Before data collection, informed consent was obtained from all participants and their guardians. Trained master's students in physical education served as on-site supervisors and provided standardized instructions during questionnaire administration.

During the data-cleaning process, invalid questionnaires characterized by consecutive identical responses, obvious logical errors, and severe missing data were first removed, and outliers were excluded based on standardized z scores to improve data quality and reduce the influence of extreme values. Ultimately, 504 valid questionnaires were retained, yielding a valid response rate of 94.9%. In terms of sample structure, there were 258 boys (51.2%) and 246 girls (48.8%), and the age of the participating students ranged from 12 to 18 years, with a mean age of 14.5 ± 2.5 years.

### Study instrument

#### Chinese version of the life skills scale for physical education

This study employed the *Chinese Version of the Life Skills Scale for Physical Education (C-LSSPE)* to assess the level of LS development among secondary school students in the context of physical education classes. The C-LSSPE was developed by Ji et al. (33). Based on the original Life Skills Scale for Sport (LSSS) and adapted to the physical education class context (5). And has an appropriate theoretical basis and contextual applicability. A large body of previous research has shown that processes such as cooperation, competition, self-expression, and reflection in physical activity may support students' LS development, and sport has also been widely recognized as an ideal vehicle for promoting adolescents' social adaptation and personal growth (33, 34). The C-LSSPE contains 43 items across eight dimensions: teamwork, goal setting, social skills, issue decision, emotional skills, leadership, time management, and communication. The item stem is “Physical education classes taught me to…”. In the present sample, the Cronbach's α coefficients of the scale dimensions ranged from 0.87 to 0.93, indicating good internal consistency. Further confirmatory factor analysis indicated acceptable model fit: χ^2^/df = 2.500, CFI = 0.930, TLI = 0.920, RMSEA = 0.055, and SRMR = 0.044, indicating that the scale demonstrated good structural validity.

#### Physical education engagement scale-student

The revised *Physical Education Engagement Scale-Student (PEES-S)* consists of 17 items and is divided into three dimensions: vitality, dedication and concentration (34). In the present study, the Cronbach's α coefficients for the three dimensions ranged from 0.82 to 0.92. With the three latent dimensions allowed to correlate, the fit indices were within acceptable ranges (χ^2^/df = 4.230, CFI = 0.94, TLI = 0.93, RMSEA = 0.079, SRMR = 0.036), indicating acceptable to good overall fit.

#### Physical environment of physical education classes satisfaction scale

The *Physical Education Class Physical Environment Satisfaction Scale* used in this study was adapted from the “physical environment” subscale of the *Questionnaire of School Physical Activity Environment (Q–SPACE)* developed by Robertson-Wilson, to better fit the context of secondary school physical education classes in China (35). To ensure linguistic accuracy and contextual suitability, the scale was translated, back- translated, and culturally adapted for Chinese secondary school physical education classes. A panel of five experts, including psychologists, sport psychologists, coaches, linguists, and psychometricians, reviewed the translated and back-translated items. The panel evaluated semantic equivalence, conceptual consistency, cultural appropriateness, and practical applicability before the scale was finalized for use in this study. The final scale contained four items assessing equipment quantity, equipment quality, venue space, and perceived teaching safety, each rated on a 5-point Likert scale. In terms of structural validity testing, AMOS was used to conduct CFA within a structural equation model (SEM), and after allowing the residuals of the items “quantity of equipment” and “quality of equipment” to correlate, the goodness-of-fit indices indicated acceptable to good model fit (χ^2^/df = 2.979, CFI = 1.000, TLI = 0.990, RMSEA = 0.063, SRMR = 0.005). The Cronbach's α coefficient for the scale was 0.92, indicating good internal consistency. Although the number of items is small, the core dimensions of the physical environment covered are highly consistent with the realities of secondary school physical education classes, and these procedures support the content validity and practical applicability of the scale (36).

### Statistical analysis

AMOS 28.0 was used to conduct confirmatory factor analysis of the scales; SPSS 25.0 was used to perform Harman's single-factor test, descriptive statistics, and Pearson correlation analysis; and the PROCESS macro v4.1 for SPSS was used to test the statistical mediation models, using 5,000 bootstrap resamples to estimate the indirect effects.

Model fit was evaluated using commonly reported indices, including χ^2^/df, CFI, TLI, RMSEA, and SRMR. PEOPEC was entered as the predictor variable, PECLE as the mediator, and the total LS score and its eight dimensions as outcome variables in separate models. The indirect effects were tested using the bootstrap method with 5,000 resamples. An indirect effect was considered statistically significant when its 95% bootstrap confidence interval did not include zero. The statistical mediation pattern was interpreted according to the significance of the indirect effect and the remaining direct association. Partial mediation was identified when the bootstrap confidence interval of the indirect effect did not include zero and the direct association between PEOPEC and LS remained statistically significant PECLE was included in the model. A full statistical mediation pattern was considered present when the bootstrap confidence interval of the indirect effect did not include zero, whereas the direct effect became non-significant after PECLE was included in the model.

## Results

### Common method bias

This study used Harman's single-factor test to assess potential common method bias (37). The results indicated that, in the unrotated factor solution, nine factors with eigenvalues greater than 1 were extracted, and the first factor explained 39.29% of the variance, which was below the 40% threshold. Therefore, it can be concluded that common method bias did not significantly affect the results of this study.

### Correlation analysis and descriptive statistics

Descriptive statistics indicated that participants reported mean scores above the scale midpoint for PEOPEC, PECLE, and LS (Table 1). Cronbach's α coefficient were calculated to assess the internal consistency of each dimension, the reliability coefficients of all dimensions across the three scales in the present analysis were found to range from 0.82 to 0.93, indicating acceptable to excellent internal consistency.

**Table 1:** Means, standard deviations, Cronbach's alpha coefficients, and Pearson correlations among the study variables.

Dimension	PEOPEC	Vitality	Ded	Con	TW	GS	SS	ID	ES	LD	TM	CS
PEOPEC 1												
Vitality	0.119^**^	1										
Ded	0.247^**^	0.761^**^	1									
Con	0.119^**^	0.805^**^	0.773^**^	1								
TW	0.320^**^	0.366^**^	0.454^**^	0.359^**^	1							
GS	0.402^**^	0.386^**^	0.477^**^	0.352^**^	0.629^**^	1						
SS	0.213^**^	0.452^**^	0.468^**^	0.424^**^	0.617^**^	0.501^**^	1					
ID	0.319^**^	0.397^**^	0.494^**^	0.400^**^	0.607^**^	0.643^**^	0.605^**^	1				
ES	0.258^**^	0.413^**^	0.472^**^	0.420^**^	0.523^**^	0.542^**^	0.584^**^	0.655^**^	1			
LD	0.294^**^	0.469^**^	0.553^**^	0.456^**^	0.752^**^	0.601^**^	0.684^**^	0.685^**^	0.648^**^	1		
TM	0.257^**^	0.443^**^	0.568^**^	0.477^**^	0.544^**^	0.586^**^	0.549^**^	0.648^**^	0.697^**^	0.683^**^	1	
CS	0.142^**^	0.355^**^	0.441^**^	0.346^**^	0.541^**^	0.424^**^	0.647^**^	0.577^**^	0.637^**^	0.685^**^	0.629^**^	1
Means	3.44	3.57	3.80	3.67	3.78	3.82	3.66	3.60	3.61	3.65	3.65	3.78
SD	1.30	1.05	0.85	0.94	0.84	0.84	0.89	0.94	0.96	0.85	0.97	0.92
Cronbach	0.92	0.92	0.82	0.90	0.93	0.93	0.87	0.91	0.90	0.92	0.90	0.90

^**^p < 0.01.

*N* = 504; at the 0.01 level (double tail), the correlation is significant. PEOPEC, Physical Environment of Physical Education Classes; Ded, Dedication; Con, concentration; TW, Teamwork; GS, Goal Setting; SS, Social Skills; ID, Issue Decision; ES, Emotional Skills; LD, Leadership; TM, Time Management; CS, Communication Skills.

### Mediating effect

The study utilized Model 4 of the PROCESS macro v4.1 to estimate statistical mediation models in order to examine the indirect associations among the study variables. Age, gender, and grade were included as covariates in all models. One overall statistical mediation model and 24 dimension-specific statistical mediation models were estimated. For presentation purposes, the 24 dimension-specific models were summarized into three figures. The first model is Model A included PEOPEC as the predictor variable, vitality as the mediator, and the eight dimensions of LS as outcome variables. Model B included PEOPEC as the predictor variable, dedication as the mediator, and the eight dimensions of LS as outcome variables. Model C included PEOPEC as the predictor variable, concentration as the mediator, and the eight dimensions of LS as outcome variables. The path coefficients shown in the figures are unstandardized regression coefficients.

**Figure 1:**
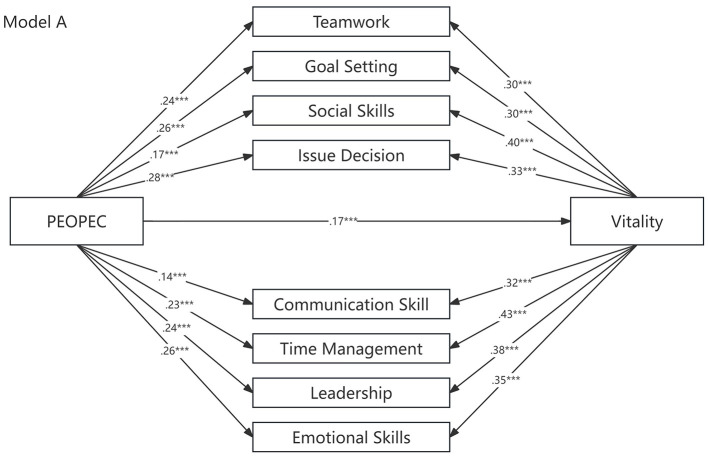
Mediation model of the vitality dimension. The regression models for vitality (Model A) is shown with the values on the lines representing the unstandardized regression coecients. Gender, age, and grade are included as covariates in all three models. ***p < 0.001.

According to the regression analysis (Figure 1), PEOPEC was significantly and positively associated with the three dimensions of PECLE (Table 2): vitality (b = 0.17, *p* < 0.001), dedication (b = 0.20, *p* < 0.001), and concentration (b = 0.18, *p* < 0.001). In terms of total effects, the total associations between PEOPEC and the eight dimensions of LS were statistically significant (b = 0.14–0.28, *p* < 0.001). Regarding the remaining direct associations, after vitality was included as the mediator, the direct association estimates between PEOPEC and the LS dimensions decreased but remained statistically significant (b = 0.09–0.22, all *p* < 0.05), indicating a partial statistical mediation pattern involving vitality. The indirect associations through vitality were statistically significant for all eight dimensions of LS with effect values as follows: teamwork [β = 0.05, 95% CI (0.02, 0.08)], GS [β = 0.05, 95% CI (0.02, 0.08)], SS [β = 0.07, 95% CI (0.04, 0.10)], ID [β = 0.06, 95% CI (0.03, 0.09)], ES [β = 0.06, 95% CI (0.03, 0.09)], LD [β = 0.07, 95% CI (0.04, 0.10)], TM [β = 0.07, 95% CI (0.04, 0.11)], and CS [β = 0.06, 95% CI (0.03, 0.09)]. PEOPEC showed statistically significant indirect associations with adolescents' LS through vitality, and the indirect association through vitality was statistically significant in the relationship between PEOPEC and LS.

**Table 2:** Regression results for the statistical mediation models with vitality as the mediator.

Model effect results			Normal theory tests			
Model	Path coefficient (b)	Normal effect	*SE*	*t*	*P*	95% CI
TW						
Total effect	0.25	0.25	0.03	7.69	0.001	[.18, 0.31]
Vitality	0.3	0.3	0.04	8.44	0.001	[.23, 0.37]
Model 1	F = 16.78^***^, R = 0.34, R^2^ = 0.11					
GS						
Total effect	0.26	0.26	0.03	8.58	0.001	[.20, 0.33]
Vitality	0.3	0.3	0.03	8.84	0.001	[.23, 0.37]
Model 2	F = 26.46^***^, R = 0.42, R^2^ = 0.18					
SS						
Total effect	0.17	0.17	0.04	4.71	0.001	[.10, 0.23]
Vitality	0.4	0.4	0.04	10.83	0.001	[.33, 0.48]
Model 3	F = 7.75^***^, R = 0.24, R^2^ = 0.06					
ID						
Total effect	0.28	0.28	0.04	7.85	0.001	[.20, 0.35]
Vitality	0.34	0.34	0.04	8.53	0.001	[.26, 0.41]
Model 4	F = 30.94^***^, R = 0.49, R^2^ = 0.24					
ES						
Total effect	0.26	0.26	0.04	7.08	0.001	[.19, 0.33]
Vitality	0.35	0.35	0.04	8.61	0.001	[.27, 0.43]
Model 5	F = 15.16^***^, R = 0.33, R^2^ = 0.11					
LD						
Total effect	0.24	0.24	0.03	7.31	0.001	[.17, 0.30]
Vitality	0.38	0.38	0.03	11.07	0.001	[.31, 0.45]
Model 6	F = 15.60^***^, R = 0.33, R^2^ = 0.11					
TM						
Total effect	0.23	0.23	0.03	6.13	0.001	[.16, 0.30]
Vitality	0.43	0.43	0.04	10.78	0.001	[.35, 0.51]
Model 7	F = 10.71^***^, R = 0.28, R^2^ = 0.08					
CS						
Total effect	0.14	0.14	0.04	3.85	0.001	[.07, 0.21]
Vitality	0.33	0.33	0.04	8.03	0.001	[.25, 0.41]
Model 8	F = 4.27^***^, R = 0.18, R^2^ = 0.03					

^***^p < 0.001.

**Figure 2:**
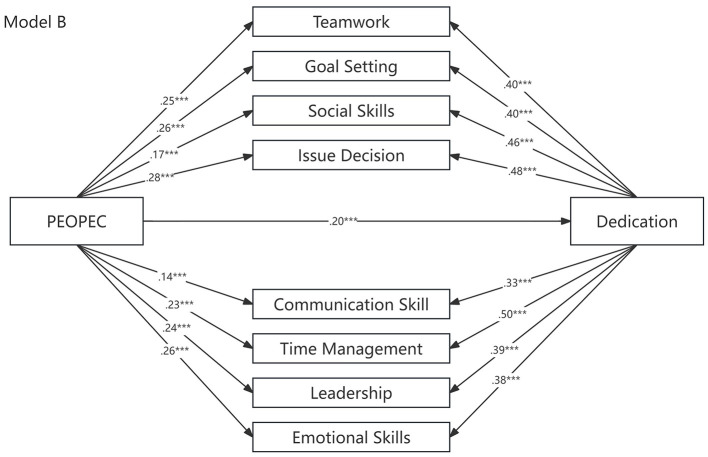
Mediation model of the dedication dimension. The regression models for vitality (Model B) is shown with the values on the lines representing the unstandardized regression coecients. Gender, age, and grade are included as covariates in all three models. ***p < 0.001.

In Model B (Figure 2), the total association estimates between PEOPEC and the eight dimensions of LS were statistically significant (b = 0.14–0.28, *p* < 0.001) (Table 3). Regarding the remaining direct associations, after Dedication was included as a mediating variable, the direct association estimates between PEOPEC and the LS dimensions decreased but remained statistically significant (b = 0.05–0.19, all *p* < 0.05), indicating that dedication played a partial mediating role. The indirect associations through dedication were statistically significant for all eight dimensions of LS, with the indirect association estimates summarized in the text. The results suggested a consistent partial statistical mediation pattern through dedication in the associations between PEOPEC and the LS dimensions, the indirect association estimates were descriptively larger than those observed for vitality in Model A.

**Table 3:** Regression results for the statistical mediation models with dedication as the mediator.

Model effect results			Normal theory tests			
Model	Path coefficient (b)	Normal effect	*SE*	*t*	*P*	95% CI
TW						
Total effect	0.25	0.25	0.03	7.69	0.001	[.18, 0.31]
Ded	0.4	0.4	0.04	9.87	0.001	[.32, 0.48]
Model 1	F = 35.52^***^, R = 0.51, R^2^ = 0.26					
GS						
Total effect	0.26	0.26	0.03	8.52	0.001	[.20, 0.33]
Ded	0.4	0.4	0.04	10.23	0.001	[.32, 0.47]
Model 2	F = 26.46^***^, R = 0.42, R^2^ = 0.18					
SS						
Total effect	0.17	0.17	0.03	4.71	0.001	[.10, 0.23]
Ded	0.46	0.46	0.04	10.53	0.001	[.38, 0.55]
Model 3	F = 29.75^***^, R = 0.48, R^2^ = 0.23					
ID						
Total effect	0.28	0.28	0.04	7.85	0.001	[.21, 0.35]
Ded	0.48	0.48	0.04	10.77	0.001	[.39, 0.57]
Model 4	F = 40.80^***^, R = 0.54, R^2^ = 0.29					
ES						
Total effect	0.26	0.26	0.04	7.08	0.001	[.19, 0.33]
Ded	0.47	0.47	0.05	10.05	0.001	[.38, 0.56]
Model 5	F = 34.77^***^, R = 0.51, R^2^ = 0.26					
LD						
Total effect	0.24	0.24	0.03	7.31	0.001	[.17, 0.30]
Ded	0.51	0.51	0.04	13.05	0.001	[.43, 0.57]
Model 6	F = 15.59^***^, R = 0.33, R^2^ = 0.11					
TM						
Total effect	0.23	0.23	0.04	6.13	0.001	[.17, 0.30]
Ded	0.62	0.62	0.04	14.12	0.001	[.54, 0.71]
Model 7	F = 10.71^***^, R = 0.28, R^2^ = 0.08					
CS						
Total effect	0.14	0.14	0.04	3.85	0.001	[.07, 0.21]
Ded	0.47	0.47	0.05	10.18	0.001	[.38, 0.56]
Model 8	F = 24.83^***^, R = 0.45, R^2^ = 0.20					

^***^p < 0.001.

**Figure 3:**
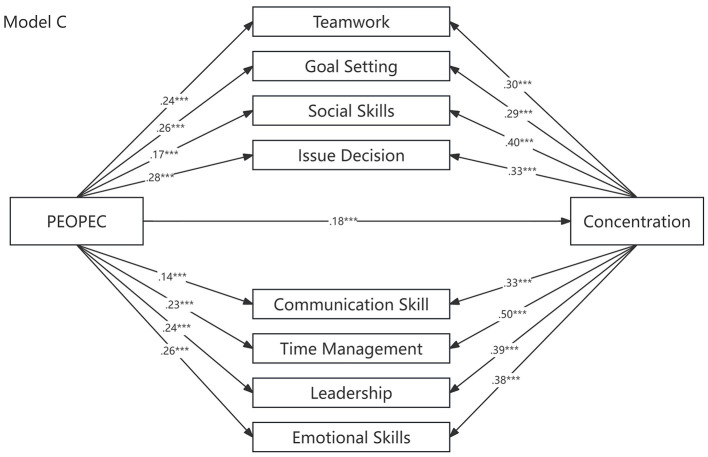
Mediation model of the concentration dimension. The regression models for vitality (Model C) is shown with the values on the lines representing the unstandardized regression coecients. Gender, age, and grade are included as covariates in all three models. ***p < 0.001.

In Model C (Figure 3), the indirect association through concentration was observed for TM (β = 0.05), which was significantly higher than that on the other dimensions (Table 4), suggesting that the indirect association through concentration was most pronounced for time management among the examined models. Regarding the remaining direct associations, after concentration was included as the mediator, the direct association estimates between PEOPEC and the LS dimensions decreased but remained statistically significant (β = 0.08–0.22, all *p* < 0.05), indicating a partial statistical mediation pattern involving concentration. The effect values for social skills (β = 0.09, *p* < 0.05) and communication skills (β = 0.08, *p* < 0.05) were the lowest, suggesting that these associations may involve additional mediators that were not examined in the present model. A comparison of Model A (vitality), Model B (dedication), and Model C (concentration) suggested differences in the magnitude of the indirect association estimates across different dimensions of PECLE. The mediating effect of concentration was most pronounced for time management, whereas Vitality and Dedication exerted stronger indirect effects on other skills, such as teamwork and leadership.

**Table 4:** Regression results for the statistical mediation models with concentration as the mediator.

Model effect results			Normal theory tests			
Model	Path coefficient (b)	Normal effect	*SE*	*t*	*P*	95% CI
TW						
Total Effect	0.25	0.25	0.04	7.69	0.001	[.18, 0.31]
Con	0.31	0.31	0.04	8.01	0.001	[.23, 0.38]
Model 1	F = 27.97^***^, R = 0.47, R^2^ = 0.22					
GS						
Total Effect	0.26	0.26	0.03	8.58	0.001	[.20, 0.33]
Con	0.29	0.29	0.04	7.78	0.001	[.22, 0.36]
Model 2	F = 35.81^***^, R = 0.51, R^2^ = 0.26					
SS						
Total Effect	0.17	0.17	0.04	4.71	0.001	[.10, 0.23]
Con	0.4	0.4	0.04	9.83	0.001	[.32, 0.48]
Model 3	F = 26.71^***^, R = 0.46, R^2^ = 0.21					
ID						
Total Effect	0.28	0.28	0.04	7.85	0.001	[.21, 0.35]
Con	0.36	0.36	0.04	8.51	0.001	[.28, 0.44]
Model 4	F = 30.83^***^, R = 0.49, R^2^ = 0.24					
ES						
Total Effect	0.26	0.26	0.04	7.08	0.001	[.19, 0.33]
Con	0.38	0.38	0.04	8.69	0.001	[.29, 0.47]
Model 5	F = 15.16^***^, R = 0.33, R^2^ = 0.11					
LD						
Total Effect	0.24	0.24	0.03	7.31	0.001	[.17, 0.30]
Con	0.39	0.39	0.04	10.43	0.001	[.32, 0.47]
Model 6	F = 36.91^***^, R = 0.52, R^2^ = 0.27					
TM						
Total Effect	0.23	0.23	0.04	6.13	0.001	[.16, 0.30]
Con	0.5	0.5	0.04	11.71	0.001	[.41, 0.58]
Model 7	F = 10.71^***^, R = 0.28, R^2^ = 0.08					
CS						
Total Effect	0.14	0.14	0.04	3.85	0.001	[.07, 0.21]
Con	0.33	0.33	0.04	7.53	0.001	[.25, 0.42]
Model 8	F = 15.15^***^, R = 0.36, R^2^ = 0.13					

^***^p < 0.001.

**Figure 4:**
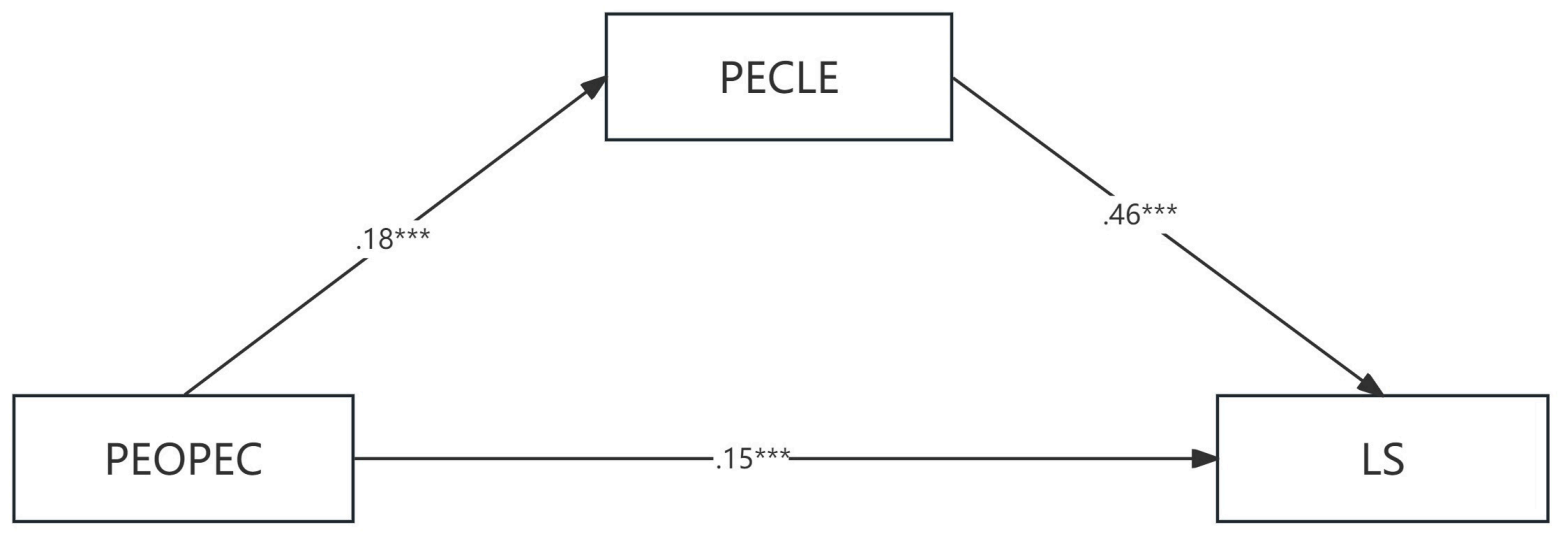
The mediation model for the overall model. ***p < 0.001.

Finally, the study examined an overall model using composite scores for PECLE and LS. According to the path coefficients of the overall model (Figure 4), PEOPEC was significantly and positively associated with LS (b = 0.23, *P* < 0.001). After PECLE was included in the model, the direct association between PEOPEC and LS decreased (β = 0.15, *P* < 0.01), but remained statistically significant, indicating partial mediation. At the same time, the path from PECLE to LS was statistically significant [b = 0.46, *p* < 0.001, 95% CI (0.38, 0.53)]. Taken together, these findings indicate that these findings supported a partial statistical mediation pattern involving PECLE.

## Discussion

### The influence of PEOPEC on LS

This study focuses on the role of PECLE in the relationship between PEOPEC and the development of LS. The results showed that, across all models, PEOPEC was significantly positively associated with LS development, which was consistent with Hypothesis H1 in the current sample. Compared with previous studies, differences in variable dimensions may explain why statistically significant associations have not been consistently observed across all eight dimensions of LS (12). In the present study, although the associations between PEOPEC and LS varied across dimensions, they were particularly pronounced in issue decision, emotional skills, and goal setting. For example, the condition and quality of equipment, may be related to students' situational experience, and may be associated with students' perceptions of environmental feedback during PE classes. It should be noted that this interpretation is still based on correlational and mediation analyses, and therefore is more appropriately understood as a hypothesized indirect association rather than a strictly identified causal mechanism. Even so, this finding is consistent with the current policy orientation in school physical education that emphasizes optimizing venues and facilities and improving classroom quality (38), and it also suggests that the physical environment may represent a potentially actionable entry point for supporting classroom participation and LS development.

In addition, existing questionnaire-based and experimental studies have suggested that LS may help adolescents cope with complex life situations and may be associated with reduced declines in physical activity levels (39, 40). The present study further highlights the relevance of PEOPEC in this process. According to bioecological systems theory, the school environment is a crucial microsystem for student development (18, 41). Whether classroom layout and resources are conducive to student interaction, whether equipment and spatial configurations are accessible and inclusive, and how efficiently the space is utilized are all factors that should be taken into account in teaching practice. Compared with previous studies that have mainly emphasized teacher support (42), motivational climate (43), or structured sport programs as correlates of LS, the present study extends this line of research by examining PEOPEC as a relevant contextual factor. Previous research has suggested that LS are often developed through repeated participation in cooperative, goal-oriented, and problem solving sport situations. The present findings are generally consistent with this view, but further suggest that the quality of facilities, equipment, space, and perceived safety may represent material conditions under which these learning experiences occur (9). Notably, the stronger associations observed for issue decision, emotional skills, and goal setting suggest that the physical environment may be more closely related to LS that may be closely related to situational feedback, task challenge, and students' perceived control during PE classes.

### The influence of PECLE on LS

Correlation and regression analyses showed that the three dimensions of PECLE were positively associated with the eight dimensions of LS, which was consistent with Hypothesis H2. The results indicated that the higher the level of students' engagement in physical education classes, the more favorable their overall level of LS development. Such engagement is reflected not only in active classroom participation, but also in a positive learning atmosphere and positive exercise-related attitudes and behaviors, and may be related to physical fitness, psychological adjustment, and social adaptation. It should be noted that the present study does not imply that high engagement necessarily leads to improvement in LS, but rather suggests that there is a positive association between the two in the present sample. Regular participation in physical education classes may provide opportunities for students to practice skills relevant to life and social situations, which may be related to the applicability and transferability of LS. This may be relevant to adolescents' self-understanding and self-efficacy when facing future challenges. Existing studies have shown that engagement in physical education learning is often divided into dimensions such as cognitive, emotional, and behavioral engagement (11, 41), with greater emphasis on intrinsic motivation and individual initiative. By contrast, the three-dimensional structure of “vitality–dedication–concentration” adopted in this study places greater emphasis on external contextual factors such as the social environment and classroom interaction and classroom interaction on engagement, is more closely aligned with the contextual characteristics of physical education classes, may further inform the theoretical understanding of engagement in physical education learning, and highlights the differentiated associations between dimensions of engagement and LS.

At the theoretical level, engagement is closely related to cognitive participation, and a high level of cognitive activation may be associated with LS; from a neuroscience perspective, enhanced engagement may be associated with a higher state of arousal and may be more likely to show autonomous learning intentions and proactive behavior (44). In physical education classes, when students participate more actively in cooperation, competitive interaction, rule implementation, and goal completion, students may have more opportunities to practice abilities such as problem solving, teamwork, and time management. However, the present study differs from earlier work in two respects. First, rather than treating engagement only as general classroom participation, this study examined engagement through vitality, dedication, and concentration, which may better capture students' energetic involvement, emotional investment, and sustained attention in PE classes (45). Second, the outcome variables were not limited to physical activity behavior or academic attitudes, but extended to multiple dimensions of LS. This comparison suggests that PECLE may represent a broader developmental process through which classroom participation may be associated with social, emotional, and self-regulatory competencies.

School physical education should place greater emphasis on supporting students' engagement to better realize the educational value of physical education. In concrete practice, it is recommended that high-quality physical education teaching be promoted in the following ways: first, by providing students with sufficient and diverse opportunities for sports participation; second, by strengthening the professional training of physical education teachers and improving their instructional design capacity; third, by establishing evidence-based evaluation and monitoring mechanisms, and fourth, by creating a safe, interesting, and vibrant physical education classroom environment (46). Such approaches may support students' engagement, sustained participation in physical education learning, and LS development.

### The mediating effect of PECLE

This study found, through regression analysis, a partial statistical mediation pattern involving PECLE in the association between PEOPEC and LS development, which was consistent with Hypothesis H3. The results showed that the remaining direct association between PEOPEC and the leadership dimension was b= 0.24, whereas the indirect effect generated through the Dedication dimension of engagement reached as high as b = 0.51. This result suggests that the association between the physical environment and leadership may be partially accounted for by students' dedication, rather than being reflected only in the remaining direct association with venues or facilities themselves. Specifically, when the sports equipment provided in class is characterized by diversity, challenge, and enjoyment, and when the design of team-based activities encourages cooperation and role-taking, students may report greater emotional and energetic involvement in collective activities, as well as stronger perceived self-worth, belonging, responsibility, and willingness to lead. This possible shift from “passive participation” to “active responsibility-taking” may reflect the relevance of classroom environments to learning dedication and students' leadership-related development. Therefore, policy recommendations should not remain only at the level of optimizing the quantity of facilities and spatial layout, but should pay greater attention to how environmental design may support students' deep learning motivation and role participation. For example, group cooperative projects, rotating team leader mechanisms, and student-led activity organization may be relevant to students' emotional engagement, sense of responsibility, and leadership-related LS. When students are enthusiastic about physical education classes and participate actively, they are more likely to adapt to and efficiently use the physical resources provided in class; conversely, if they lack learning motivation, they may be less attentive to these environmental elements, which may be associated with less favorable learning experiences. This finding is consistent with previous studies (47). The richness of teaching content and the diversity of equipment may be associated with student participation and a positive classroom atmosphere; abilities practiced in the physical education environment may be transferable to non-sport settings in order to advance individual development, and skill practice and knowledge acquisition are components of students' broader development (48).

In summary, there is a close relationship among PEOPEC, PECLE, and LS. To support students' holistic development, schools may focus on two aspects: optimizing the teaching environment and supporting classroom engagement—in order to provide students with richer, more diverse, and more challenging learning experiences. More importantly, the skills acquired in the physical education environment should be transferable to non-physical-education settings, which may be relevant to students' sustained cognitive, emotional, and social responsibility development. Physical education classes provide adolescents with authentic situations for practicing communication, teamwork, problem solving, and perseverance, and these experiences may help them overcome fear, build self-confidence, and support the integration and application of LS (49).

## Limitation

This study also has certain limitations, particularly the relatively narrow theoretical focus on the mechanisms underlying LS. At present, only a simple statistical mediation pattern among PEOPEC, PECLE, and LS was examined, whereas possible additional mediators or moderated mediation pathways were not considered. Future longitudinal or experimental studies are needed to clarify the temporal and causal relationships among PEOPEC, PECLE, and LS. In terms of sampling, different school types, such as urban and rural schools, were not clearly distinguished or evenly represented, and the regional coverage was limited to the central and eastern regions of Hubei Province. To a certain extent, this limits the analysis and generalization of differences in PEOPEC across different regions and across different school contexts, and limits the development of targeted recommendations for optimizing PEOPEC. Future studies may consider expanding the sample size and incorporating more regions and school types so as to enhance the representativeness and generalizability of the findings. The reliance on self- report questionnaires may have introduced common method bias and inflated the associations among the study variables. Future studies should incorporate multi-source data, such as teacher ratings, classroom observations, and objective assessments of the PE environment, to improve the validity of the findings. Because there is currently no scale specifically designed to assess PEOPEC in the Chinese secondary school context, an adapted version of an existing physical environment subscale was used in this study. Although the scale underwent translation, back-translation, cultural adaptation, expert review, and preliminary psychometric testing, further psychometric validation is still needed in future studies.

## Conclusion

This study tested a statistical mediation model linking PEOPEC, PECLE, and LS. The results indicated that students who perceived a more favorable PEOPEC tended to report higher levels of LS, and this association was partially statistically mediated by PECLE. The results indicated that a favorable PEOPEC was positively associated with multidimensional LS, and this association was partially statistically mediated by PECLE.

The study further emphasizes that optimizing the physical education classroom environment is an important means of improving students' classroom participation and the transfer of LS. In educational practice, attention should be paid to the systematic design of factors such as teaching space layout, equipment configuration, and safety assurance, so as to stimulate students' exercise motivation and classroom concentration. Teachers should also attend to the positive role of environmental construction in guiding PECLE, thereby promoting the full realization of the educational value of physical education curricula.

## Data Availability

The original contributions presented in the study are included in the article/supplementary material, further inquiries can be directed to the corresponding author/s.
